# The Roles of c-Jun N-Terminal Kinase (JNK) in Infectious Diseases

**DOI:** 10.3390/ijms22179640

**Published:** 2021-09-06

**Authors:** Jing Chen, Chao Ye, Chao Wan, Gang Li, Lianci Peng, Yuanyi Peng, Rendong Fang

**Affiliations:** 1 Joint International Research Laboratory of Animal Health and Animal Food Safety, College of Veterinary Medicine, Southwest University, Chongqing 400715, China; cjing1235@163.com (J.C.); yechao123@swu.edu.cn (C.Y.); w10241229@163.com (C.W.); li18438695575@163.com (G.L.); penglianci@swu.edu.cn (L.P.); 2 Immunology Research Center, Medical Research Institute, Southwest University, Chongqing 402460, China; 3 Chongqing Key Laboratory of Herbivore Science, Chongqing 400715, China

**Keywords:** JNK, apoptosis, autophagy, infectious diseases, JNK inhibitors

## Abstract

c-Jun N-terminal kinases (JNKs) are among the most crucial mitogen-activated protein kinases (MAPKs) and regulate various cellular processes, including cell proliferation, apoptosis, autophagy, and inflammation. Microbes heavily rely on cellular signaling pathways for their effective replication; hence, JNKs may play important roles in infectious diseases. In this review, we describe the basic signaling properties of MAPKs and JNKs in apoptosis, autophagy, and inflammasome activation. Furthermore, we discuss the roles of JNKs in various infectious diseases induced by viruses, bacteria, fungi, and parasites, as well as their potential to serve as targets for the development of therapeutic agents for infectious diseases. We expect this review to expand our understanding of the JNK signaling pathway’s role in infectious diseases and provide important clues for the prevention and treatment of infectious diseases.

## 1. Introduction

**Figure 1:**
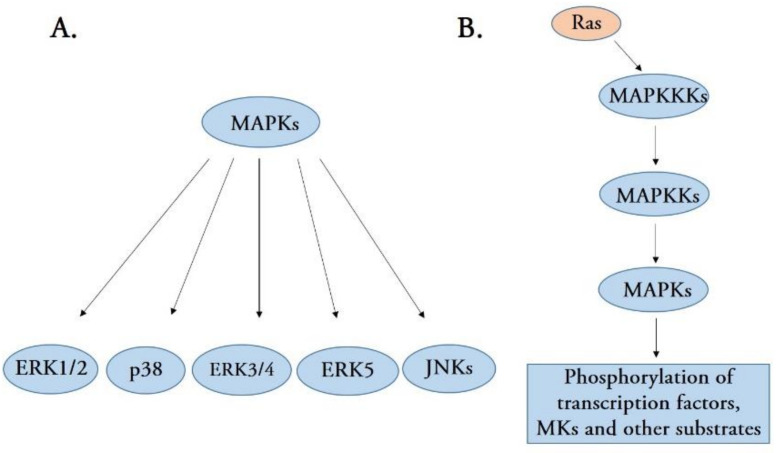
(**A**) A schematic representation of the five distinct groups of mitogen-activated protein kinases (MAPKs), including extracellular signal-regulated kinases 1 and 2 (ERK1/2), p38, ERK3/4, ERK5, and c-Jun N-terminal kinases (JNKs). (**B**) Stress-activated MAPK signaling pathways. MAPK signaling pathways are structurally organized as a signaling cascade, leading to the phosphorylation of many transcription factors.

Mitogen-activated protein kinases (MAPKs), a new serine/threonine kinase, comprise an intracellular signaling cascade network that activates when cells recognize and respond to various extracellular stimuli [1]. MAPK signaling pathways have been found in all eukaryotic cells and are involved in diverse biological functions, including gene expression, mitosis, cell growth, migration, and survival [2]. Currently, five distinct groups of MAPKs have been identified in mammals, namely extracellular signal-regulated kinases 1 and 2 (ERK1/2), p38 MAPK isoforms, ERK3/4, ERK5, and c-Jun N-terminal kinases (JNKs) [1] (Figure 1A). Although each group of MAPKs has unique features, each MAPK member shares a set of evolutionarily conserved and sequentially acting kinases (a MAPK, a MAPK kinase (MAPKK), and a MAPKK kinase (MAPKKK)) [3]. The first activated kinase from these three components is MAPKKK, which activates by phosphorylation or interaction with a small GTP-binding protein of the Ras/Rho family in response to extracellular stimuli [4]. MAPKKK activation leads to the phosphorylation and activation of MAPKK, which activates MAPK activity by phosphorylating the threonine and tyrosine residues in the MAPK activation loop [5,6]. Therefore, MAPKs are the final kinases in the MAPK cascade and phosphorylate substrates at serine and threonine residues. Most of the substrates for MAPK are cellular transcription factors additionally, several other substrates can also be phosphorylated by MAPKs, including phospholipases, cytoskeletal proteins, and several protein kinases termed MAPK-activated protein kinases (MKs) (Figure 1B). MKs mediate a wide range of biological functions, including the regulation of gene expression, mRNA stability and translation, cell proliferation, and survival [1,2,3].

c-Jun N-terminal kinases (JNKs) represent the MAPK family and play critical roles in balancing cell survival and death in response to both extracellular and intracellular stresses [7]. JNKs are also known as stress-activated protein kinases (SAPKs) because they exhibit robust activity under cellular stress induced by bacterial toxins, environmental stressors, and proinflammatory cytokines [8]. Therefore, JNK signaling may play critical roles in many biological diseases by regulating various cellular processes, including inflammatory responses, differentiation, proliferation, death, and survival [8,9]. JNK proteins consist of JNK1, JNK2, and JNK3, encoded by the three distinct genes MAPK8, MAPK9, and MAPK10, respectively [10]. Furthermore, the three JNK proteins are cleaved into at least ten alternatively spliced variants: four JNK1 isoforms, four JNK2 isoforms, and two JNK3 isoforms [11]. JNK1 and JNK2 are ubiquitously expressed in body tissues, whereas JNK3 is restricted to only the brain, heart, and testis [9,12]. Although JNK2 exhibits an amino acid identity of 83% and similar regulatory functions to JNK1, it binds c-Jun (a main substrate of JNKs) more efficiently than JNK1 [13], suggesting that the two kinases execute both similar and different biological responses. JNK1 and JNK2 have been known to play crucial roles in obesity and diabetes [14,15], immune disorders, cancer progression [16], and various respiratory diseases [17]. Predominantly expressed in the brain, JNK3 is considered a potential therapeutic target for neurodegenerative diseases [18]. Given that JNK proteins are highly responsive to various extracellular stimuli, diseases involving JNKs are not restricted to those listed above. Recent studies have suggested that JNKs play crucial roles in various infectious diseases, including viral, bacterial, fungal, and parasitic infections [19,20,21,22].

**Figure 2:**
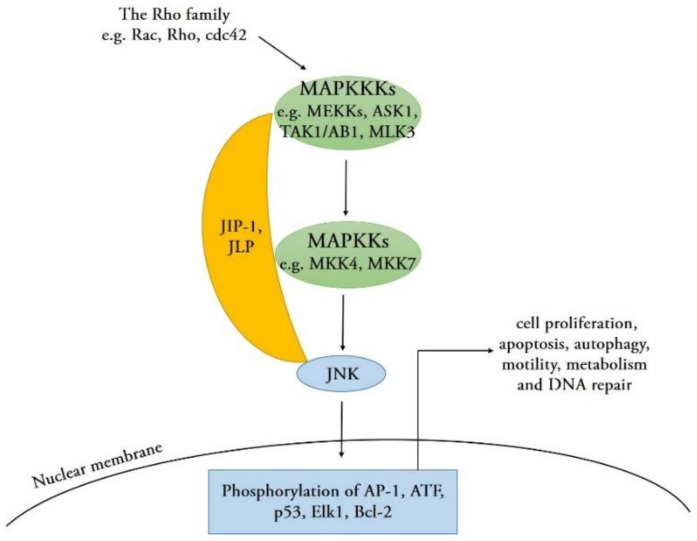
Mechanisms for the activation of the JNK signaling pathway. These MAPK kinase kinase (MAPKKK) proteins are activated by small GTPases (Rac, Rho, and cdc42) of the Rho family, which activate in response to various stress stimuli. JIP-1 and JLP act as scaffold proteins that bind the upstream kinases MAPKKK, MAPK kinase (MAPKK), and the MAPK (JNK) into a specific signaling module. Subsequently, JNK is activated and translocated into the nucleus and phosphorylates specific substrates, such as activator protein 1 (AP-1), activating transcription factor (ATF), p53, Elk1, and B-cell lymphoma 2 (Bcl-2). The activation of these downstream substrates regulates a wide range of cellular transcription profiles and subsequently induces numerous cellular processes.

Comparable to other members of the MAPK family, JNKs are activated by upstream MAPKKs (MKK4 and MKK7) via phosphorylation on threonine and tyrosine residues within their conserved Thr-Pro-Tyr (TPY) motif [1]. MAPKKs are phosphorylated and activated by several upstream MAPKKKs, such as MEKKs, ASK1, TAK1/AB1, or MLK3. These MAPKKK proteins are activated by small GTPases of the Rho family (Rac, Rho, and cdc42) that activate in response to various stress stimuli, such as environmental stresses, inflammatory cytokines, GPCR agonists, and growth factors [9]. In addition, scaffold proteins such as JNK-interacting proteins (JIPs) and JNK-associated leucine zipper proteins (JLPs) combine with various components in JNK signaling pathways to coordinate the signaling network [23]. Upon activation, the JNK protein translocates into the nucleus and phosphorylates serine and threonine residues on specific substrates, such as the transcription factor activator protein-1 (AP-1) family proteins, activating transcription factors (ATF), p53, ETS Like-1 protein (Elk1), and cell death regulators, such as those of the Bcl-2 family [9,24]. The activation of these downstream substrates regulates a wide range of cellular transcription profiles; subsequently, it induces numerous cellular processes and defense responses, including cell proliferation, apoptosis, autophagy, motility, metabolism, and DNA repair [25] (Figure 2), which may have an important regulatory effect on the development of various biological and infectious diseases.

This review aims to summarize the roles of JNKs in cellular signaling transduction and infectious diseases caused by microbes. We hope this review elucidates the relationship between JNK signaling and various infectious diseases and emphasizes the potential of JNKs to serve as targets for the development of therapeutic agents for infectious diseases.

## 2. JNK Signaling in Apoptosis and Autophagy

Many studies have indicated that all three JNKs are involved in stimulating apoptotic cell death. Initially, delayed and persistent JNK1 activation was highly correlated with apoptosis induced by exposure to gamma radiation [26]. A study by Tournier et al. later defined the unequivocal requirements of JNK1 and JNK2 for UV-induced apoptosis in primary murine embryonic fibroblasts (MEFs). MEFs lacking JNK1/2 showed resistance to apoptosis in response to UV radiation [27]. Similarly, JNK3 may be critical for stress-induced neuronal apoptosis; the apoptotic role of JNK3 was confirmed using JNK3^−/−^ mice showing a reduction in hippocampal neuron apoptosis induced by glutamate [28].

Two distinct mechanisms are currently involved in JNK-mediated apoptosis. In one mechanism, phosphorylated JNK is translocated to the nucleus upon activation, where it phosphorylates c-Jun and other transcription factors. Then, JNK promotes apoptosis by increasing the transcription of known pro-apoptotic genes, such as Fas/FasL signaling pathway-related genes, via the transactivation of c-Jun/AP-1-dependent or p53-dependent mechanisms. Finally, the binding of FasL to Fas mediates the activation of caspase 8, which further activates the downstream effector caspase 3 to trigger apoptosis [29,30]. In another mechanism, activated JNK translocates to mitochondria where it phosphorylates Bcl-2/Bcl-xL and antagonizes the anti-apoptotic activity of Bcl-2/Bcl-xL. Moreover, JNK expression leads to the release of cytochrome C via a Bid-Bax-dependent mechanism, activating caspase 9 and caspase 3 and inducing apoptosis [30]. In summary, it has been suggested that the JNK pathway plays a positive role in stress-induced apoptosis.

The JNK signaling pathway has also been reported to participate in autophagy regulation in response to various stress signals. For instance, a previous study showed that JNK1, but not JNK2, is required for starvation-induced autophagy [31]. By contrast, JNK2, but not JNK1, activates pro-survival autophagy and inhibits palmitic acid lipotoxicity during palmitic acid-induced autophagy [32]. In addition, compared with starvation- and palmitic acid-induced autophagy, oncolytic adenovirus-mediated autophagy is promoted by both JNK1 and JNK2 isoforms [33]. Since JNK3 is only expressed in the brain, heart, and testis, the role of JNK3 in inducing autophagy is rarely reported. To date, JNK has been thought to modulate autophagy through several distinct mechanisms. In the first mechanism, JNK promotes the phosphorylation of the Bcl-2/Bcl-xL and therefore interferes with its ability to bind to the pro-autophagy BH3 domain-containing protein Beclin 1; this rapidly results in the dissociation of Beclin 1 from the Beclin 1-Bcl-2/Bcl-xL complex via a Bcl2L11 (also known as BIM) phosphorylation-dependent mechanism. The freed Beclin 1, which is required to initiate autophagosome formation, stimulates autophagy [34]. In addition, JNK also phosphorylates several transcription factors, including AP-1 family members c-Jun, Fos, and FoxO, which mediate the transcription of autophagic genes such as Beclin 1. In the last mechanism, JNK activation also leads to the upregulation of the damage-regulated autophagy modulator (DRAM), a p53 target gene. The overexpression of DRAM promotes the accumulation of autophagosomes by regulating the autophagosome-lysosome fusion leading to autolysosome formation [25,35]. Overall, the JNK signaling pathway plays a key role in the autophagy process and promotes autophagy initiation in a variety of ways.

## 3. Role of JNK Signaling Pathway in Viral Diseases

**Figure 3:**
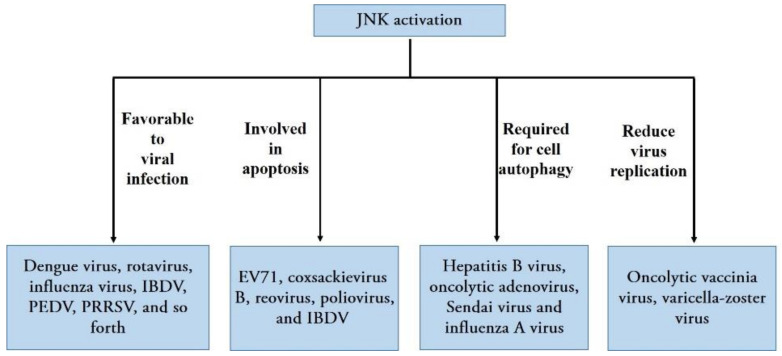
The different roles of JNK activation in regulating various viral infections; these include facilitating or reducing virus replication for different types of viruses and triggering apoptosis or autophagy during each virus infection.

Viruses are a group of strictly intracellular parasites that invade the cells of virtually all known organisms; they use the host cell’s machinery to replicate effectively, produce new progeny, and generally damage or kill the host cell in the infection process. Viruses cause a wide variety of human, animal, and plant diseases that negatively affect human health, breeding, and planting industries [36]. The long-term coevolution of viruses and their hosts has led to the host cell responding to viral infection by activating various cellular signaling pathways; these host signaling molecules may support or inhibit viral replication [19]. JNK, an important component in MAPK signaling, becomes activated by diverse groups of viruses [37] and regulates many viral infections in different ways (Figure 3).

Data from previous research have shown that a variety of viruses can manipulate JNK signaling to regulate their replication, i.e., RNA viruses including human immunodeficiency virus type 1 (HIV-1) [38], echovirus 1 [39], and DNA viruses, such as the herpes simplex virus type 1 [40], Kaposi’s sarcoma-associated herpesvirus [41], and varicella-zoster virus (VZV) [42]. Generally, the activation of the JNK signaling pathway upon viral infection favors viral infection. JNK activation is essential for effective viral replication, and inhibiting JNK activation leads to reduced viral replication according to studies on several human viruses, such as dengue virus [43], rotavirus [44], and influenza virus [45]; veterinary viruses, including infectious bursal disease virus (IBDV) [37], porcine epidemic diarrhea virus (PEDV) [8], porcine reproductive and respiratory syndrome virus (PRRSV) [46], and white spot syndrome virus, a type of dangerous aquatic virus [47] (Figure 3). Moreover, it appears that JNK regulates viral replication and infection at different levels. A recent report showed that the bovine ephemeral fever virus triggers Src-JNK-AP1 signaling pathways at the stage when the virus binds to induce cellular clathrin and dynamin 2 expression; the activation of Src-JNK-AP1 subsequently facilitates virus entry in an autocrine or paracrine fashion [48]. Moreover, the activation of JNK occurs early in dengue virus infection, and the interaction between a UV-inactivated virion and the host cell surface molecules triggers JNK phosphorylation, JNK phosphorylation, and activation, which are presumably involved in the entry and early infection process of the dengue virus [43]. However, UV-irradiated inactivated PEDV failed to induce the phosphorylation of JNK1/2, suggesting that viral biosynthesis is essential for activating these JNKs during the PEDV infection. Furthermore, inhibiting JNK1/2 activation results in a significant reduction in viral RNA synthesis, protein expression, and PEDV progeny release, suggesting that JNK activation may be required for viral RNA replication and gene expression [8]. For poliovirus, however, the inhibition of JNK activation delayed cell death and viral release without affecting poliovirus replication; therefore, JNK may play a role in early poliovirus release [49] (Figure 3).

Numerous viruses have evolved distinct mechanisms to activate JNK signaling by exploiting their encoded proteins. For instance, the NS1 protein of most influenza A virus subtypes can activate JNK, and the amino acid positioned at 103 in NS1 plays a critical role in this signaling event [50]. The Epstein–Barr virus-encoded latent membrane protein 1 (LMP1) induces JNK activation through its C-terminal activating region 2 (CTAR2) [51]. A study on herpes simplex virus demonstrated that ICP27 protein was sufficient and necessary for JNK activation [52]; furthermore, the Tat protein of HIV-1 activates JNK MAP kinases through an oxidant-dependent mechanism [53,54]. However, neither the downstream activators that these viral proteins directly interact with, nor their direct effects have been well-elucidated. In the future, more in-depth research must be performed to explore the molecular mechanisms by which other viral-encoded proteins are used to activate JNK in detail.

Apoptosis and autophagy are two highly regulated forms of programmed cell death in mammalian cells that control homeostasis-related cell growth and the cellular immune response to invading pathogens [25,45,55]. Consistent with apoptosis, autophagy plays vital roles in cell death, normal physiology, and cellular homeostasis. However, autophagy has been shown to play a dual role in protecting and killing stressed cells. Both autophagy and apoptosis may be triggered by shared upstream signals, resulting in the activation of combined or exclusive autophagy and/or apoptosis [25]. Many studies have recently suggested that the JNK pathway plays a key role in both apoptotic cell death and autophagy induced by viral infections. Initially, the activation of the JNK pathway was found to be involved in apoptosis induced by several viruses. For example, evidence has indicated that the JNK pathway mediates apoptosis induced by enterovirus 71 (EV71) [56], coxsackievirus B3 [57], reovirus [58], poliovirus [49], and IBDV [37]. In addition, JNK participates in inducing apoptosis in infected cells and is required for viral infection and replication, which enhances viral replication in most cases [37,59,60,61]. In addition, JNK activation is required for cell autophagy induced by different types of viruses, such as hepatitis B virus [62], oncolytic adenovirus [63], Sendai virus [64], and influenza A virus [45] (Figure 3). Notably, the inhibition of JNK signaling not only significantly inhibits virus-induced autophagosome formation, but also suppresses the replication of the corresponding virus. Therefore, JNK signaling is critical for viral replication via the induction of autophagy; this is consistent with reports indicating that viruses positively manipulate autophagy for effective replication and host cell lysis [65].

Although JNK activation enhances viral replication among a variety of viruses, JNKs plays an important role in inhibiting the replication of some viruses. For example, JNK controls oncolytic vaccinia virus replication through PKR pathway activation, and JNK deficiency can significantly enhance oncolytic vaccinia virus replication [66]. Similarly, the use of inhibitors demonstrated that inhibiting JNK resulted in a two-fold increase in VZV replication, whereas the constitutive activation of JNK resulted in a decline in VZV replication [67]. Moreover, the inhibition of JNK increases VZV replication in melanoma cells but decreases VZV replication in fibroblasts, suggesting that the role of JNK in VZV pathogenesis depends upon the type of cell infected [68] (Figure 3).

## 4. The Role of JNK Signaling in Bacterial, Fungal and Parasitic Infections

In addition to viruses, bacteria are the second most common cause of infectious diseases. Infection by bacterial pathogens and the host’s defense against infection is a lifetime battle between bacteria and the host, where a combination of host defense mechanisms, including MAPK pathways and some virulence factors of invaded bacterial pathogens, are involved [69].

JNK has been shown to activate at higher levels upon bacterial infection in multiple mammalian cell types [70,71]. Recent studies have suggested that JNKs also play crucial roles in the innate immunity of invertebrates. JNK homolog (ChJNK) expression in oysters upregulated significantly upon infection with *Vibrio alginolyticus* and *Staphylococcus haemolyticus*, suggesting that ChJNK may be involved in the host’s defense against bacterial infection [72]. A similar result was observed in a study that exposed the Yesso scallop to gram-positive and gram-negative bacteria [73]. These findings indicate a high activation rate for JNK in the immune defense of both vertebrate and invertebrate organisms.

**Figure 4:**
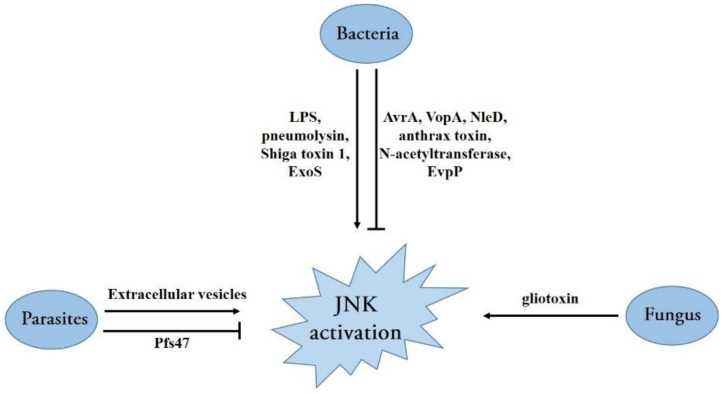
Diagram of various mechanisms of bacteria, fungus, and parasites activating or disrupting the JNK pathway.

Bacteria have evolved various mechanisms to activate the JNK signaling pathway (Figure 4). For instance, bacterial lipopolysaccharide (LPS), a conserved component of the outer membrane of a gram-negative bacterium, has been shown to initiate JNK activation in monocyte/macrophage cells, and other types of cells [74,75,76]. Several cell surface proteins, such as CD14, CD36, TLR4, and MD-2, have been reported to initiate a downstream signaling pathway inducing JNK activation [74,76,77]. Moreover, pneumolysin, a potent pneumococcal virulence factor, induces ATF3 expression via the activation of TLR4 and JNK pathways and protects from *Streptococcus pneumoniae* infection by activating cytokines [78]. Shiga toxin 1, produced by the Shiga toxin-producing *Escherichia coli* (*E. coli*), triggers a ribotoxic stress response depending on its enzymatic activity, leading to JNK activation and induction in apoptosis for intestinal epithelial cells [79]. The opportunistic bacterial pathogen *Pseudomonas aeruginosa*-encoded ExoS, a virulence factor produced and secreted directly into the host cell by the type III secretion system (T3SS), also activates JNK phosphorylation and triggers cellular apoptosis depending on the subsequent JNK-mediated signaling [80] (Figure 4).

In addition, many bacteria have evolved strategies to manipulate and subvert host MAPK signaling through translocated effector proteins that directly catalyze the post-translational modification of proteins in the MAPK network [81] (Figure 4). The AvrA protein in *Salmonella typhimurium* is an important *Salmonella* effector protein secreted by *the Salmonella* T3SS that directs acetyltransferase activity toward specific host MAPKKs, inhibiting signaling through the host JNK/AP-1 pathway and dampening the pro-apoptotic innate immune response [82]. Similarly, *Vibrio parahaemolyticus* employs the T3SS effector VopA (an acetyltransferase) to modify serine, threonine, and lysine residues in MKKs (MKK1 and MKK6); consequently, all three MAPK signaling cascades including the JNK pathway are abolished [83]. By contrast, enteropathogenic and enterohemorrhagic *E. coli* transfer the effector protein NleD (a zinc metalloprotease) into host cells via the T3SS; this directly cleaves JNK and p38 between the Gly/Pro and Tyr of the TPY motif, rendering them inactive [84]. The causative agent of anthrax *Bacillus anthracis*, as a gram-positive bacterium, secretes a major virulence factor: anthrax toxin. Similar to NleD, the lethal factor subunit of anthrax toxin is a zinc metalloprotease that cleaves the N-terminus of several MKKs, including MKK1-4 and MKK6-7, resulting in the permanent inactivation of these kinases. The inactivation of these kinases also leads to abolishing the ERK1/2, p38, and JNK signaling pathways [85,86,87]. *Mycobacterium tuberculosis*, the pathogen causing tuberculosis, secretes an enhanced intracellular survival protein (an efficient N-acetyltransferase) that activates the JNK-specific phosphatase DUSP16 through the acetylation of Lys55. Therefore, JNK phosphorylation is abolished by increased levels of dephosphorylation, facilitating pathogen survival inside cells by reducing cell death and inflammation [88] (Figure 4). Although an increasing number of bacterial effector mechanisms have been revealed in recent years, many effectors with unknown mechanisms are yet to be discovered, representing a major challenge to future research.

Recently, several studies showed that JNKs play critical roles in the activation of inflammasomes. It was reported that JNK1 directly phosphorylates NLRP3 at S194, which is a critical priming event and essential for NLRP3 inflammasome activation [89]. JNK was also required to activate ASC-containing inflammasomes as JNK regulates ASC phosphorylation at Y144 [90,91]. Furthermore, TAK1 was confirmed to be an upstream kinase of JNK, and the activation of the TAK1-JNK pathway is critical to ASC speck formation [92]. Given the importance of inflammasome activation in controlling bacterial infection, it is not surprising that bacteria have evolved various strategies to counteract inflammasome activation. For instance, a novel T6SS effector, EvpP in *Edwardsiella tarda*, can target intracellular Ca^2+^ signaling to impair JNK activation and subsequent ASC-containing inflammasome activation [93] (Figure 4).

Opportunistic fungal infections are among the leading causes of death among immune-compromised patients, posing a threat to human health. Although few studies have been conducted on the interaction between the JNK pathway and pathogenic fungi, it was recently found that MAPK pathways are predominantly affected, with increased levels of phospho-p38 and phospho-JNK in the infection model of *Trichophyton equinum* [94]. Moreover, the mold *Aspergillus fumigatus*, which causes invasive aspergillosis in immune-compromised patients, exploits its major virulence factor, gliotoxin, to activate the JNK pathway and subsequently induce apoptosis in a Bim EL phosphorylation-dependent manner [95] (Figure 4). In addition, the role of JNK activation in host antifungal responses has been extensively studied. JNK1 induced by *Candida albicans* can negatively regulate host antifungal innate immune responses in vivo by suppressing CD23 expression [21]. Therefore, JNK plays a critical role in fungal infection, indicating that JNK may be a therapeutic target for fungal treatment.

Although pathogenic protozoan parasites are not as harmful as virulent bacteria and viruses, evidence suggests that some parasites cause diseases in humans and animals, either alone or in conjunction with other pathogens. Similar to other infections, many parasite infections can result in MAPK signaling pathway activation; JNK activation has been reported in the infection process of *Theileria parva* [96], *Toxoplasma gondii* [97], *Trypanosoma cruzi* [98], *Plasmodium berghei* [99], and *Neospora caninum* [100]. Furthermore, it was demonstrated that extracellular vesicles secreted by *Neospora caninum* are rapidly internalized into host cells, where they activate JNK signaling in a TLR2-dependent manner [100] (Figure 4). The role of JNK in host resistance and pathology during *Toxoplasma gondii* infection has been investigated. JNK2 plays a role in *Toxoplasma gondii*-induced immunopathology and simultaneously promotes host susceptibility to this pathogen [101]. Interestingly, Pfs47 expressed by *Plasmodium falciparum* in the mosquito stages can disrupt mosquito JNK signaling and the activation of apoptosis-associated caspases, rendering the parasites “invisible” to the mosquito’s immune system and thus increasing its survival and chances of transmission through the mosquito vector [102] (Figure 4). Presumably, these parasites have evolved different strategies to activate or inactivate the JNK pathway at different stages of infection to ensure their survival. In the future, more in-depth work must elucidate the role of JNK in parasitic infection.

## 5. JNK Signaling Inhibitors as Therapeutic Agents against Infectious Diseases

The JNK signaling pathway is closely related to the initiation and development of various biological diseases. The development of selective JNK inhibitors that target specific JNK-mediated pathological diseases is promising. Currently, JNK is considered a potential therapeutic target for many diseases. Numerous JNK pathway inhibitors have been entered into preclinical and clinical trials as potential therapies for diabetes, cancer, depression, neurotrauma, hearing loss, and even Alzheimer’s disease, exhibiting a future perspective in the context of many disease therapies [9,15,103,104,105,106].

A host of infectious diseases can pose major threats to humans and animals, and there are often no special drugs to prevent these diseases. Regarding viruses, novel species, such as SARS-CoV-2, which caused the worldwide COVID-19 pandemic, are continually emerging, and vaccines are often ineffective in preventing these infections [107]. Hence, a novel, alternative, and specific therapy is urgently needed to control viral infections. Recently, increasing evidence suggests that JNK is a promising therapeutic target in the context of infectious diseases. JNK-specific inhibitors, such as ATP-competitive inhibitors and small peptide inhibitors, have been used for suppressing JNK activation in infectious disease-related studies (Table 1).

In these studies, SP600125 is the most widely used ATP-competitive inhibitor. SP600125 binds to the ATP-binding site in all known JNKs, preventing their phosphorylation and activation [108]. Recent studies have evaluated the effects of SP600125 in models for a variety of pathogenic infections, especially viral infections. Most of these studies indicated that the SP600125 treatment significantly decreased the replication or production of each pathogen, such as the Kaposi’s sarcoma-associated herpesvirus [41], human cytomegalovirus [109], rotavirus [44], fowl adenovirus serotype 4 [110], bovine herpesvirus 1 [111], epizootic hemorrhagic disease virus [112], several *Orthopoxvirus* species [113], the bacterial agent *Brucella melitensis* [114] in vitro, and even the fungus *Candida albicans* in vitro and in vivo [21]. Furthermore, a few studies have demonstrated that stimulating SP600125 can inhibit the cell entry of the West Nile virus [115], hepatitis C virus [116], and *Chlamydia pneumonia* [117]. By contrast, in rare cases, SP600125 treatment enhanced the replication and production of some pathogens, as seen in the oncolytic vaccinia virus [66]. In addition, although uncommon, the replication of several pathogens, including the Japanese encephalitis virus [118], coxsackievirus B3 [119], vesicular stomatitis virus [120], PRRSV [121], and poliovirus [49], appears to be unaffected by SP600125. Other ATP-competitive inhibitors, such as AS601245 and JNK-IN-8, have also been used in several JNK-related studies; they both showed inhibitory activity against the hepatitis C virus [116] and respiratory syncytial virus [122]. However, these inhibitors have varying degrees of toxicity and lack specificity because they indiscriminately inhibit the phosphorylation of all JNK substrates [123].

Several small peptide inhibitors targeting the protein–protein interactions of JNK and its substrates, including c-Jun and adaptor proteins such as JNK-interacting protein (JIP), have been developed; for example, JNK peptide inhibitor 1 and JIP1 [104,124] have demonstrated their ability to suppress the replication and production of porcine circovirus type 2 [125] and herpes simplex virus type 1 [126], respectively. These novel inhibitors are not selective inhibitors of JNK1-3. Therefore, novel JNK isoform-specific inhibitors must be developed in the future. In addition, these conclusions are based on in vitro experimental investigations, and whether these results comport with future clinical trials remains to be determined.

## 6. Conclusions

The recent emergence of the SARS-CoV-2 virus and the subsequent pandemic have caused unprecedented healthcare and economic problems worldwide. Meanwhile, the continued prevalence and threat of COVID-19 also exposes the limitations of vaccine efficacy and the lack of more effective antiviral therapies. Hence, developing new antiviral strategies is highly urgent and desirable. Targeting host cell pathways that support the virus and microbial replication can directly inhibit the infection of various microbes; therefore, this approach is a promising anti-infection therapy.

The JNK signaling pathway regulates many cellular events, including the cell cycle, differentiation, survival, apoptosis, and inflammatory responses. Viruses, bacteria, fungi, and parasites exploit various cellular processes and events to ensure their survival and cause infectious diseases in humans, animals, and plants. Viruses and other pathogens have been shown to lead to JNK activation and maintain successful infections in a JNK-dependent manner. Therefore, JNK can be considered a potential therapeutic target for many infectious diseases. However, the understanding of JNK functions in various infectious diseases is still limited and complicated. JNK activation exhibits different effects in different pathogens and shows diametrically opposite effects on the same pathogen. Hence, when selecting JNK as a target for treating infectious diseases, its effect as a suppressor or promoter must be considered. In addition, widely used JNK inhibitors currently contain a certain degree of toxicity and lack the required specificity for each JNK isoform. Researchers must explore more specific and less toxic inhibitor molecules, and clinical trials at different stages must be advanced. Although challenging, there remains a bright future for JNK inhibitors and their application in therapies for infectious diseases, particularly in important viral diseases.

## Figures and Tables

**Table 1:** Summary of JNK inhibitors studied in infectious diseases.

Type of Inhibitor	Inhibitor	Effects on Pathogens	Pathogens	References
ATP-competitive inhibitors	SP600125	Reduce pathogen replication	Viruses: Kaposi’s sarcoma-associated herpesvirus, human cytomegalovirus, rotavirus, fowl adenovirus serotype 4, bovine herpesvirus 1, epizootic hemorrhagic disease virus, Orthopoxviruses. Bacteria: *Brucella melitensis* Fungus: *Candida albicans*	[21,41,44,109,110,111,112,113,114]
		Inhibit pathogen entry	Viruses: West Nile virus, hepatitis C Virus Bacteria: *Chlamydia pneumonia*	[115,116,117]
		Enhance pathogen replication	Viruses: Oncolytic vaccinia virus	[66]
		No significant effects	Viruses: Japanese encephalitis virus, coxsackievirus B3, vesicular stomatitis virus, porcine reproductive, respiratory syndrome virus, and poliovirus.	[49,118,119,120,121]
	AS601245	Reduce pathogen replication	Viruses: hepatitis C Virus	[116]
	JNK-IN-8	Reduce pathogen replication	Viruses: respiratory syncytial virus	[122]
Small peptide inhibitors	JNK peptide inhibitor 1	Reduce pathogen replication	Viruses: porcine circovirus type 2	[125]
	JIP-1	Reduce pathogen replication	Viruses: herpes simplex virus type 1	[126]
